# Fatal Early-onset Sepsis Caused by Intrauterine Transmission of Serogroup Y Meningococcus

**DOI:** 10.1097/INF.0000000000003722

**Published:** 2022-09-28

**Authors:** Niek B. Achten, Jasper V. Been, Sam Schoenmakers, Clementien L. Vermont, Robert M. Verdijk, Irwin K. M. Reiss, H. Rob Taal

**Affiliations:** From the * Department of Pediatrics, Erasmus MC-Sophia Children’s Hospital, University Medical Centre Rotterdam, Rotterdam, The Netherlands; † Division of Neonatology, Department of Pediatrics, Erasmus MC-Sophia Children’s Hospital, University Medical Centre Rotterdam, Rotterdam, The Netherlands; ‡ Division of Obstetrics, Department of Obstetrics and Gynaecology, Erasmus University Medical Centre Rotterdam, Rotterdam, The Netherlands; § Division of Infectious Diseases & Immunology, Department of Pediatrics, Erasmus MC-Sophia Children’s Hospital, University Medical Centre Rotterdam, Rotterdam, The Netherlands; ¶ Department of Pathology, Erasmus University Medical Center Rotterdam, Rotterdam, The Netherlands.

**Keywords:** early-onset sepsis, meningococcus, infection, transmission

## Abstract

Infections by meningococcal species are extremely rare in the first days of life. We present a fatal case of early-onset sepsis presenting at birth, caused by intrauterine transmission of serogroup Y *N. meningitidis*, evidenced clinically and histologically by corresponding chorioamnionitis and *N. meningitidis*-positive amniotic fluid. This case confirms a long-standing suspicion that *N. meningitidis* can be transmitted in utero.

Intrauterine infection or inflammation of fetal membranes, amniotic fluid, or umbilical cord, summarized by chorioamnionitis, is a well-known pathogenic mechanism for fetal infection, preterm birth, and early-onset sepsis (EOS) of the infant.^1,2^ Notorious pathogens are group B *Streptococcus* and *E. coli*^3^; less frequent are enterococcus or staphylococcus species. Infections by meningococcal species, however, are extremely rare in the first days of life. We present a fatal case of early-onset sepsis presenting perinatally caused by intrauterine transmission of *N. meningitidis*, serogroup Y, proving a clinically important and previously hypothesized mechanism.^4,5^

## CASE REPORT

Following an uncomplicated pregnancy, a 26-year-old pregnant woman (gravida 2, para 1, not vaccinated for type Y *N. meningitidis*), presented at exactly 29 weeks of gestation with ruptured fetal membranes for half a day, vaginal discharge of green-colored fluid, and uterine contractions. She reported a 2-week history of headache, urticaria, and elevated temperature up to 37.8°C. Her vaginal discharge during pregnancy had increasingly become more green-colored over the last month. At presentation to our center, her laboratory infection markers were elevated (Table 1), and cardiotocography showed a fetal tachycardia (180 to 200 beats per minute) and minimal beat-to-beat variability. Due to imminent premature delivery, betamethasone was administered for lung maturation. Because of suspected intrauterine infection, tocolysis was not initiated, while intravenous amoxicillin/clavulanic acid was started approximately 1 hour prior to delivery. Given the progression of labor, vaginal delivery was considered the quickest and safest obstetrical management plan.

**TABLE 1.** Maternal Laboratory Results

Laboratory Test	Value	Reference Range
Hemoglobin, g/dL	12.1	12.1–15.3
MCV, fL	90	80–100
Platelets, ×10^9^/L	333	150–370
Leukocytes, ×10^9^/L	23	3.5–10.0
CRP, mg/L	15	< 10

Laboratory results from blood tests drawn 2 hours before delivery.

CRP indicates C-reactive protein; MCV, mean cell volume.

Spontaneous vaginal delivery occurred within 2 hours after admission, with remarkably green-colored amniotic fluid. A male infant (birthweight 1590 g [93th percentile, Fenton curve]), was born with a bradycardia of <60 beats per minute. Umbilical cord blood showed a metabolic acidosis (Table 2). After insufflation ventilations, electrocardiogram monitoring showed a heart rate increasing to >100 beats per minute. Fraction of inspired oxygen (FiO_2_) was increased to 1.0 because of peripheral oxygen saturation (SpO_2_) below target range. Apgar scores were 1, and 6 after 1 and 5 minutes, respectively. Despite adequate ventilation and FiO_2_ of 1.0, the patient remained below target SpO_2_ and was intubated. Despite this, the patient clinically deteriorated with bradycardia, and cardiopulmonary resuscitation was initiated including chest compressions and administration of epinephrine and a fluid bolus. Severe systemic inflammatory response syndrome caused by sepsis was suspected; a blood culture was obtained and empiric antibiotics (penicillin/gentamicin) were administered in the delivery room. Surfactant was administered endotracheally, with no clear effect on SpO_2_. After in-house transfer to the neonatal intensive care unit, high-frequency oscillation ventilation with FiO_2_ 1.0 and nitric oxide was initiated because of suspected pulmonary hypertension. Intensive inotropic treatment using epinephrine and norepinephrine was initiated. At 45 minutes after birth, a second episode of chest compressions and two epinephrine doses were needed because of bradycardia despite adequate ventilation. Laboratory results showed severe metabolic and respiratory acidosis, coagulopathy, and a pattern of severe acute inflammatory response highlighted by interleukin-6 (IL-6) levels of >2.5 million pg/mL (Table 2). A chest radiogram showed signs of grade III respiratory distress syndrome. The patient’s clinical and biochemical condition deteriorated rapidly. Following consultation of multiple senior members of the team and discussion with the parents, the decision was made to withdraw intensive care, after which the patient deceased. Parents did not opt for autopsy.

**TABLE 2.** Neonatal Laboratory Results

Laboratory Test	Umbilical Cord Blood		Postpartum Blood Sample	
	Value	Reference Range	Value	Reference Range
pH	7.10	7.35–7.45	6.77	7.31–7.45
pCO_2_, kPa	8.6	4.7–6.4	11.9	4.0–6.4
pO_2_, kPa	<1.3	10.0–13.3	7.1	5.4–12.4
Base excess, mmol/L	−10	−3 to 3	−22	−3 to 3
Lactate, mmol/L	9.3	0.5–1.7	16.0	0.8–1.5
Hemoglobin, mg/dL			10.0	13.5–20.1
MCV, fL			120	91–106
Platelets, ×10^9^/L			142	144–449
Leukocytes, ×10^9^/L			3.8	8.0–15.4
APTT, seconds			>180	21–33
PT, seconds			53.1	11.2–15.5
Fibrinogen, g/L			1.0	1.3–3.3
Factor V, U/mL			0.14	0.82–1.45
D-dimers, mg/L			1.83	<3.49
CRP, mg/L			2.9	<10
PCT, mg/L			>100.00	<0.046
IL-6, pg/L			2,568,520	<10

Results from (arterial) umbilical cord blood and blood drawn 30 minutes (blood gas and lactate), and approximately 1 hour (hematology, coagulation, infection parameters) after birth.

APTT indicates activated partial thromboplastin time; CRP, C-reactive protein; IL-6, interleukin-6; MCV, mean cell volume; PCT, procalcitonin; PT, partial thromboplastin time.

**FIGURE 1.**
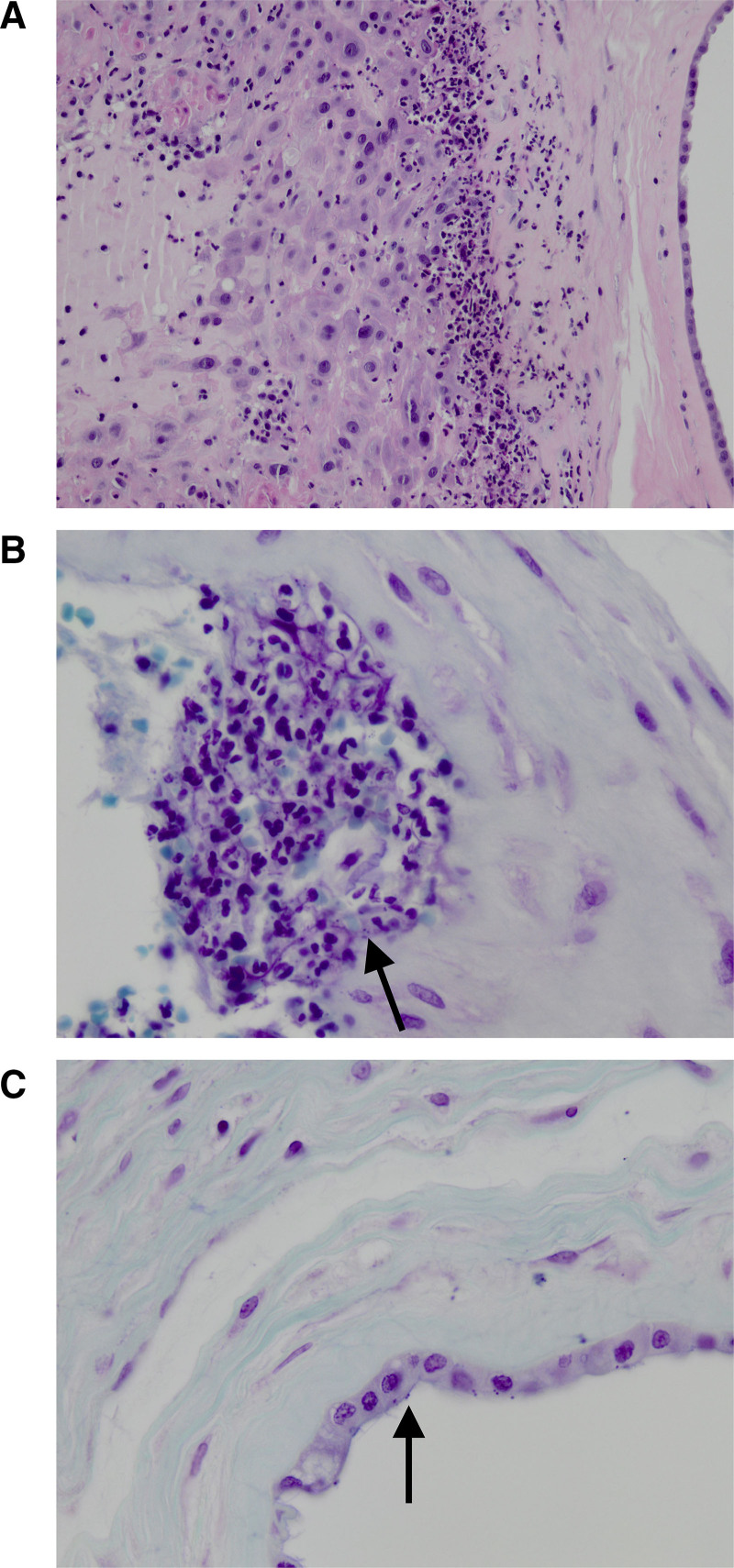
Histopathology of the placenta showing severe chorioamnionitis with presence of Gram-negative diplococci. A: Chorioamnionitis of the membranes showing neutrophil infiltration of both the chorion and amnion (Hematoxylin and eosin stain 200×). B: Gram-negative cocci with occasional diplococci in the chorion (Gram stain 630×). C: Gram-negative diplococci in the amnion indicating infection of the amniotic fluid (Gram stain 630×).

Within 24 hours after birth, a blood culture obtained before start of antibiotics was positive for *N. meningitidis*, which, following further serotyping was identified as serogroup Y, expressing types 5-2 and 10-1 PorA porines and type 4-1 of the FetA membrane protein.^6^ Phenotypically, the organism was sensitive to penicillin (minimal inhibitory concentration [MIC] ≤0.047 mg/L), cefotaxime (MIC <0.002 mg/L), ciprofloxacin (MIC not quantified), and rifampicin (MIC 0.008 mg/L). Genetic analysis revealed a type 22 allele for penicillin sensitivity, reported as not clearly associated with either penicillin sensitivity or insensitivity. *N. meningitidis* was also cultured from amniotic fluid collected at presentation, without further determination of serogroup. A maternal vaginal swab for group B *Streptococcus* obtained at admission was negative. Histologic analysis of the placenta revealed severe chorioamnionitis, with abundant neutrophils in the membranes and chorionic plate (Fig. 1), but no signs of funisitis or vasculitis in the chorionic plate. Focal Gram-negative cocci, including diplococci, were identified in the placenta, most likely representing *N. meningitidis*. A final diagnosis of in utero transmitted fulminant meningococcal early-onset sepsis was established.

Following the culture results, secondary prevention was initiated in collaboration with municipal health services. Health care workers and family members considered at-risk due to immediate contact with the patient were prescribed chemoprophylaxis consisting of ciprofloxacin or an adequate alternative.

## DISCUSSION

The case described above is, to our knowledge, the first documented EOS case caused by serogroup Y *N. meningitidis* with corresponding acute chorioamnionitis. This provides important evidence for in utero transmission of *N. meningitidis* and its ability to cause fatal perinatal infection. Although EOS is dominated by group B *Streptococcus* and *E. coli*, this case underlines that the range of etiological pathogens is diverse, especially among preterm or very-low-birthweight infants.^7,8^ Although no cerebrospinal fluid was analyzed, the exceptional high levels of IL-6 in this case may be reflecting severity of disease as well as the involvement of the leptomeninges, which appear to respond to *N. meningitidis* with major IL-6 production.^9^

Neonatal *N. meningitidis* infections are rare.^4^ A 2003 review identified only 11 published cases of neonatal *N. meningitidis* infections globally, involving A, C and W serogroups if determined.^10^ Of these, eight cases presented within 3 days of birth, with a mortality rate of over 50%. Since then, few other neonatal cases have been published, including two with a serogroup Y isolate.^11–13^ Vertical (mother-to-child) transmission of *N. meningitidis* been suspected based on timing of onset of symptoms,^5,10,12–14^ similarities in genetic sequences of isolates from mother and child,^5^ and placental swab results.^14^ Vertical transmission of infectious agents can happen intrauterine, during birth, or after birth.^15^ In our case, severe histological chorioamnionitis was present, with microscopic identification of Gram-negative diplococci, and *N. meningitidis* was cultured from both amniotic fluid and the infant’s blood culture. This case therefore confirms the pathological pathway of intrauterine transmission unequivocally. Whether this intrauterine transmission occurred descending (transplacental), or ascending (through vaginal tract) is unclear: mild systemic maternal symptomatology and involvement of the chorionic plate may suggest the first, whereas the changes in maternal vaginal discharge may suggest the latter. Vaginal colonization and subsequent neonatal infection of serogroup Y *N. meningitidis* is rare but has been described.^13^

Meningococcal disease epidemiology is volatile, differs geographically, and is amended by immunization.^16^ In the United States, infections with serogroup Y *N. meningitidis* have decreased after introduction of a MenACWY vaccine in 2005,^17^ but there are concerns about recent vaccine uptake during adolescence.^18^ In Europe, serogroup Y infections were historically uncommon but have become more prevalent in the recent decade.^19^ Given the novelty and limited uptake of MenACWY vaccination, a large proportion of fertile women are still susceptible to meningococcal disease, and herd immunity is potentially still at a low level. Although currently not specifically offered as maternal vaccination, MenACWY vaccination appears safe in pregnancy,^20,21^ and the Centers for Disease Control and Prevention of the US recommend the administration of routine MenACWY vaccination during pregnancy.^22^

The presented case illustrates that midwives, obstetricians, and neonatologists should consider potential meningococcal infection in case of signs of intrauterine infection, and treat neonates with appropriately dosed therapy if it is suspected. Although *N. meningitidis* is extremely rare as a cause of EOS, it is likely preventable by adequate vaccination, unlike most other EOS pathogens. The current case therefore not only finally confirms a long-standing suspicion of mode of transmission for this pathogen, but also warrants further exploration of possibilities to improve MenACWY vaccination uptake during adolescence and perhaps pregnancy. Finally, extremely high IL-6 levels in neonates should warn neonatologists and general pediatricians of fulminant disease, and perhaps unconventional pathogens.
